# A pilot study of non-invasive diagnostic tools to detect *Helicobacter pylori* infection and peptic ulcer disease

**DOI:** 10.1038/s41598-023-50266-2

**Published:** 2023-12-20

**Authors:** En-Chih Liao, Ching-Hsiang Yu, Jian-Han Lai, Ching-Chung Lin, Chih-Jen Chen, Wen-Han Chang, Ding-Kuo Chien

**Affiliations:** 1 Department of Medicine, MacKay Medical College, New Taipei City, Taiwan; 2 Institute of Biomedical Sciences, MacKay Medical College, New Taipei City, Taiwan; 3 Department of Emergency Medicine, MacKay Memorial Hospital, Taipei, Taiwan; 4 Division of Gastroenterology, Department of Internal Medicine, MacKay Memorial Hospital, Taipei, Taiwan; 5 Department of Nursing, MacKay Junior College of Medicine, Nursing, and Management, Taipei, Taiwan; 6 Department of Medical Research, MacKay Memorial Hospital, Taipei, Taiwan; 7 Graduate Institute of Injury Prevention and Control, Taipei Medical University, Taipei, Taiwan; 8 Institute of Mechatronic Engineering, National Taipei University of Technology, Taipei, Taiwan

**Keywords:** Gastroenterology, Medical research

## Abstract

*Helicobacter pylori* (*H. pylori*) infection can lead to various digestive system diseases, making accurate diagnosis crucial. However, not all available tests are equally non-invasive and sensitive. This study aimed to compare the efficacy of non-invasive and invasive diagnostic tools for *H. pylori* infection and assess their correlation with esophagogastroduodenoscopic (EGD) findings. The study utilized the Campylobacter-Like Organism (CLO) test, serum anti-HP IgG blood test, and C-13-urea breath test (UBT) to diagnose *H. pylori* infection. A total of 100 patients with peptic ulcer symptoms, including 45 males and 55 females, were recruited for the study. Symptomatic patients between the ages of 20–70, eligible for EGD examination, were enrolled. Each diagnostic test and any combination of two positive tests were considered the reference standard and compared against the other diagnostic methods. Additionally, the relationship between these diagnostic tests and EGD findings was evaluated. Among the participants, 74.0% were diagnosed with peptic ulcer disease through EGD. The UBT demonstrated the highest Youden's index, ranging from 58 to 100%, against all the non-invasive tests. The IgG blood test displayed the highest sensitivity at 100%, with a specificity of 60–70%. On the other hand, the CLO test exhibited the highest specificity at 100% and a sensitivity of 50–85%. Furthermore, only the CLO test showed a significant association with esophageal ulcers (*p*-value = 0.01). The IgG blood test holds promise as a primary screening tool due to its exceptional sensitivity. While the UBT is relatively expensive, its non-invasive nature and high sensitivity and specificity make it a potential standalone diagnostic test for *H. pylori* infection. Moreover, the noteworthy negative correlation between the CLO test and esophageal ulcers provides evidence of the differing effects of *H. pylori* infection on antral-predominant and corpus-predominant gastritis.

## Introduction

*Helicobacter pylori* infection is a common and worldwide distributed infection^1^, which resides in the stomach, colonizes gastric epithelium, and causes digestive system diseases. *Helicobacter pylori* can colonize in the human gastric mucosa which causes mucosal defects by penetrating through the muscularis mucosae. The release of ureases raises the pH level in the stomach, thus creating an optimum environment for bacterial growth. The bacteria of *H. pylori* can be detected in the stomach, but also the dental plaque, saliva, tonsils, and even adenoid tissue related to gastroesophageal reflux. Unneglectable impacts of oral-to-oral or oral-to-fecal transmissions could widely spread through a large crowd of asymptomatic carriers^2^. Therefore, the identification of *H. pylori* plays an important role in the understanding of the pathogenesis and deterioration of diseases produced by this pathogen. Several diagnostic methods utilizing invasive or noninvasive techniques are developed to detect *H. pylori* infection^3^. The selection of diagnostic methods depends on the clinical conditions, proficiency of the clinician, sensitivity, specificity, and cost.

The complexity of dietaries and microorganisms’ infections in the gastrointestinal (GI) tract gave rise to the stomach or duodenal ulcers with dullness, sharp pain, or burning sensation^4^, most of these unendurable symptoms were pointed to the *H. pylori* infection. The susceptibility difference of *H. pylori t*o host relies on this pathogen to overcome the hostile interior milieu, leading to varying severity levels of infections. Peptic ulcer diseases (PUD) often happened in the stomach, proximal duodenum, esophagus, or Meckel's diverticulum^5^. In Asian countries, the prevalence of peptic ulcer disease (PUD) caused by *H. pylori* is notably high, estimated at approximately 93%^6^. *Helicobacter pylori* infection is not only associated with PUD but also implicated in the development of duodenal ulcers, gastric cancer, and MALT lymphoma^7–9^.

Although Taiwan is an industrialized country with an advanced medical system, the prevalence of *H. pylori* infection in Taiwan is as high as 50–60%^10^. With an increasing awareness of the pertinent features of *H. pylori* in peptic ulcer disease (PUD), clinical physicians are actively seeking the optimal diagnostic approach. In Taiwan, the consensus for *H. pylori* detection is based on invasive methods, such as endoscopic gastric biopsy with rapid urease test (also known as the Campylobacter-like organism test or CLO test) and histological analysis. Non-invasive methods, including the urea breath test (UBT) and stool antigen test (SAT), are also widely utilized. These non-invasive approaches not only accurately detect *H. pylori* infection but also facilitate monitoring of treatment outcomes without the need for recurrent invasive procedures^11^.

These tests detect *H. pylori* antigens or metabolites in breath or stool samples, serving as indicators of eradication success or treatment failure^12^. Particularly advantageous for post-treatment follow-up, they provide a reliable and cost-effective means to assess treatment response over time^11^. Easily conducted in outpatient settings, non-invasive tests are well-tolerated, in stark contrast to invasive methods such as endoscopic biopsy. The latter can be discomforting, expensive, and pose potential risks^13^.

The accuracy of non-invasive diagnostic tests for *H. pylori* infection is influenced by patient-related factors and technical considerations. Advanced age and the use of medications such as proton pump inhibitors (PPIs) or antibiotics can impact test precision. Elderly individuals, due to potential alterations in immune responses, may experience reduced reliability in serological tests. Concurrent medication use, especially PPIs and antibiotics, can contribute to false-negative results. It is crucial to take these factors into consideration when interpreting test outcomes^14,15^. Technical aspects, particularly sample collection, play a vital role in diagnostic accuracy. Suboptimal methods, insufficient samples, or processing delays can introduce discrepancies. Adherence to standardized protocols not only minimizes variability but also optimizes accuracy^16^. Taking these factors into consideration enables the precise interpretation of non-invasive diagnostic test results, mitigating the risk of false outcomes and enhancing overall accuracy. This, in turn, supports informed patient management decisions.

Patient preferences play a crucial role in selecting diagnostic tests for *H. pylori* infection and peptic ulcer disease. Considerations such as comfort, convenience, and personal preferences should be considered. Patient satisfaction and compliance are vital aspects. Non-invasive tests, such as the Urea Breath Test (UBT) or serological tests, often enjoy higher acceptability compared to invasive methods, mitigating discomfort and anxiety. Generally well-tolerated, these tests involve minimal inconvenience, leading to increased satisfaction and compliance^17^. Convenience is another crucial factor, and patients prefer tests that are easy to administer, provide quick results, and require minimal preparation. Non-invasive tests utilizing breath, blood, or stool samples offer faster turnaround times and simpler requirements, enhancing patient convenience and adherence. Considering patient preferences allows tailoring the diagnostic approach, promoting satisfaction, engagement, and compliance for positive health outcomes^18,19^.

Nevertheless, each diagnostic test has its features and limitations, which have been evaluated in numerous studies^20–23^. For instance, the serological test is unable to distinguish between acute and past infections^3^. The C-13-urea breath test (UBT) exhibits low accuracy in cases of atrophic gastritis, intestinal metaplasia, and gastric cancer. Moreover, false-positive results may arise due to urea hydrolysis by oral bacteria. The main weakness of the CLO test lies in the requirement for obtaining gastric specimens^3,17,24^. Thus, there is a necessity of evaluating the accuracy and advantage of these tools despite the American College of Gastroenterology’s Guideline on the Management of *H. pylori* Infection pointed out that no single test can be considered the gold standard for the diagnosis of *H. pylori*^25^.

### Aim

This study aims to compare three current diagnostic tools for *H. pylori* infection, including the CLO test, UBT, and IgG blood test. The objective is to correlate the results of these diagnostic tests with esophagogastroduodenoscopy (EGD) findings and interpret the relationship between them. Additionally, evaluating the correlation between diagnostic tests for *H. pylori* infection and EGD findings will contribute to achieving convergent results, facilitating better clinical consultations and decision-making.

## Materials and methods

### Study subjects and protocol of enrollment

This prospective observational study was approved by the Institutional Review Board of MacKay Memorial Hospital (18CT052be), Taipei, Taiwan. A total of 100 patients with peptic ulcer symptoms were recruited in this study, including 45 males and 55 females. Symptomatic patients aged between 20 and 70 with EGD indication were enrolled. Patients who were below 20 or above 70 years of age, experiencing out-of-hospital cardiac arrest, pregnant, undergoing hemodialysis, refusing enrollment, planning to transfer to another hospital, or had taken proton-pump inhibitors (including lansoprazole and pantoprazole) or antibiotics within the previous 2 weeks were excluded from the study. Additionally, individuals presenting with hematemesis, tarry stool, or a history of gastrectomy were also excluded.

Participant physicians would well explain this study project to patients who match the inclusion criteria. After consent was signed, the researcher would collect the patient’s baseline exhaled sample, then the patient drank 80 mL of 13C-urea reagent. After 25 min, the exhaled sample would be collected again. EGD and IgG blood tests would be done later by the schedule.

### Diagnostic tests for* H. pylori* infection

In our study, we utilized the DiaSorin LIAISON® *H. pylori* IgG Test (REF 318,980) commercial kit. As per the instruction manual, the test demonstrates a sensitivity of 95.5% (95% confidence interval: 90.4–98.4%) and a specificity of 99.2% (95% confidence interval: 97.9–99.8%). The kit employs an Indirect Chemiluminescence Immunoassay (CLIA), leveraging the specific binding properties between antibodies and antigens to immobilize the *H. pylori* antigen onto magnetic microparticles (solid phase). In the initial incubation period, *H. pylori* antibodies present in the serum will bind to the antigen on the magnetic microparticles. Subsequently, in the second incubation period, a solution containing an isoluminol derivative conjugated to anti-human IgG (a mouse monoclonal antibody to human IgG, referred to as isoluminol-Ab-Conjugate) is introduced. This solution binds to the *H. pylori* IgG attached to the magnetic microparticles. After each incubation, an additional step involves a washing procedure to remove unbound substances. Subsequently, the Start Kit, consisting of two bottles—one containing Catalyst with 4% NaOH, and the other a 0.12% H_2_O_2_ solution serving as the chemiluminescent substrate—is added. The concentration of *H. pylori* IgG is then determined by measuring the emitted chemiluminescent quantity in Relative Light Units (RLU) using a photomultiplier tube.

EGD was operated on by gastroenterological endoscopic specialists, and the following findings would be labeled on the final report: gastroesophageal reflux, esophageal ulcer, gastric ulcer, duodenal ulcer, and if the patient had one of the ulcerative findings (esophageal, gastric, and duodenal), peptic ulcer disease would be labeled. One to three biopsy samples were taken for erosive or inflammatory lesions for the CLO test. Additionally, breath samples were analyzed by Hope Wang Enterprises CO., LTD., Taipei, Taiwan, for the Urea Breath Test (UBT).

### Patient and public involvement

Patients and the public were not involved in the application of the research grants, the study design, the participant recruitment, and the conducting results of this research. No patient advisers were involved in our study. The clinical staff explained the implementation content of this research to the participating patients and obtained consent and signed documents. The clinical staff talked over the objective and concerns about this study with participating patients, including the benefit of how it will add to a better understanding of their disease etiology and triggering factors. They were notified regarding the research targets and parameters to be measured before starting the study. Patients acknowledged the consequences and usefulness of this study and allowed us to acquire and utilize their data. All the laboratory and clinical data were reported to the study participants and concluded the study findings.

### Statistical analysis

SPSS Statistics 24.0 for Windows (SPSS Inc., Chicago, IL) was used for data management and statistical analysis. Each test and any two positive tests were set as the standard and then compared against the others to calculate the sensitivity, specificity, and Youden’s index, which means when UBT was taken as the standard, the patients with UBT positive would be regarded as diseased, then IgG blood test and CLO test were calculated against UBT. Moreover, their concordances were evaluated by Cochran’s Q test because all of them were categorical variables, then Bonferroni’s test was used to correct the P-value.

We also set each EGD finding as to the outcome and evaluated the sensitivity, specificity, and Youden’s index of each diagnostic test. Pearson's chi-squared test was also performed for diagnostic tests and EGD findings, if the P-values were less than 0.05, binary logistic regression would be manipulated.

### Ethics approval and consent to participate

All procedures used in this research were approved by the Institutional Review Board of MacKay Memorial Hospital (18CT052be) and written informed consent was obtained from all subjects for publication of this information. This research was conducted following the stipulations of the Declaration of Helsinki for experiments involving humans.

## Results

### Demographic characteristics of the subjects

This study included a total of one hundred adult patients who experienced epigastric discomfort and underwent esophagogastroduodenoscopy (EGD) under physician guidance due to suspected peptic ulcer disease. Table 1 presents the demographic characteristics, showing patient ages ranging from 22 to 66 years, with 45.0% being male. Gastroesophageal reflux was observed in more than half of the patients (56.0%, 56/100), while the prevalence of peptic ulcer disease was 74% (74/100). Among the diagnosed cases, gastric ulcers were the most common (66.2%, 49/74), followed by esophageal ulcers (48.6%, 36/74) and duodenal ulcers (12.2%, 9/74).

**Table 1:** The demographic characteristics of the subjects who were enrolled in this study.

	Mean ± SD or number (%)
Age	49.51 ± 12.65
Sex	
Male	45 (45%)
Female	55 (55%)
EGD finding	
Gastroesophageal reflux disease	56 (56%)
Peptic ulcer disease	74 (74%)
Gastric ulcer	49 (49/74, 66.2%)
Esophagus ulce Esophageal ulcer	36 (36/74, 48.6%)
Esophagus ulce e Duodenal ulcer	9 (9/74, 12.2%)

*EGD* esophagogastroduodenoscopy, *SD* standard deviation.

### Head-to-head comparisons of each diagnostic test

The calculated parameters comparing each diagnostic test and even two-positive test are illustrated in Table 2. In the CLO test, it exhibited the highest specificity (100%) against each test. However, its sensitivity ranged from 51.0 to 86.7%, resulting in a decreased Youden's index. In IgG Blood Test: it showed an almost opposite pattern compared to the CLO test. It had the highest sensitivity (100%) but the lowest specificity (66.2–70.0%). In the Urea Breath Test (UBT), it yielded the highest Youden's index with the CLO test (0.946) and both tests (1.000) as the non-invasive diagnostic tests. The IgG blood test demonstrated a significantly different P-value (< 0.001) compared to all other tests, indicating heterogeneity between the IgG blood test and the other diagnostic tests. Table 3 indicates that age had no impact on the results of the IgG blood test (P = 0.118), CLO test (P = 0.792), UBT (P = 0.922), and both tests (P = 0.922). This suggests that age did not influence the diagnostic accuracy of these tests for detecting the condition being studied.

**Table 2:** Calculated parameters of each diagnostic test against non-invasive diagnostic tests.

	Test	IgG	CLO	UBT	Two positive
Sensitivity	IgG		100.0%	100.0%	100.0%
	CLO	51.0%		86.7%	86.7%
	UBT	58.8%	100.0%		100.0%
Specificity	IgG		66.2%	70.0%	70.0%
	CLO	100.0%		100.0%	100.0%
	UBT	100.0%	94.6%		100.0%
Youden's Index	IgG		0.662**	0.700**	0.700**
	CLO	0.510**		0.867	0.867
	UBT	0.588**	0.946		1.000

*IgG* immunoglobulin G, *CLO* Campylobacter-like organism; *UBT* urea breath test.

*P < 0.05; **P < 0.001.

**Table 3:** Comparisons between each diagnostic test and age.

Test	Mean ± SD of age	P value
IgG		
Positive	51.45 ± 11.48	0.118
Negative	47.49 ± 13.58	
CLO		
Positive	50.08 ± 11.02	0.792
Negative	49.31 ± 13.24	
UBT		
Positive	49.70 ± 10.61	0.922
Negative	49.43 ± 13.50	
Two positives		
Positive	49.70 ± 10.61	0.922
Negative	49.43 ± 13.50	

*IgG* immunoglobulin G, *IgG* Campylobacter-like organism, *UBT* urea breath test, *SD* standard deviation.

*P < 0.05; **P < 0.001.

### Venn diagram of each diagnostic test for *H. pylori* infection

**Figure 1:**
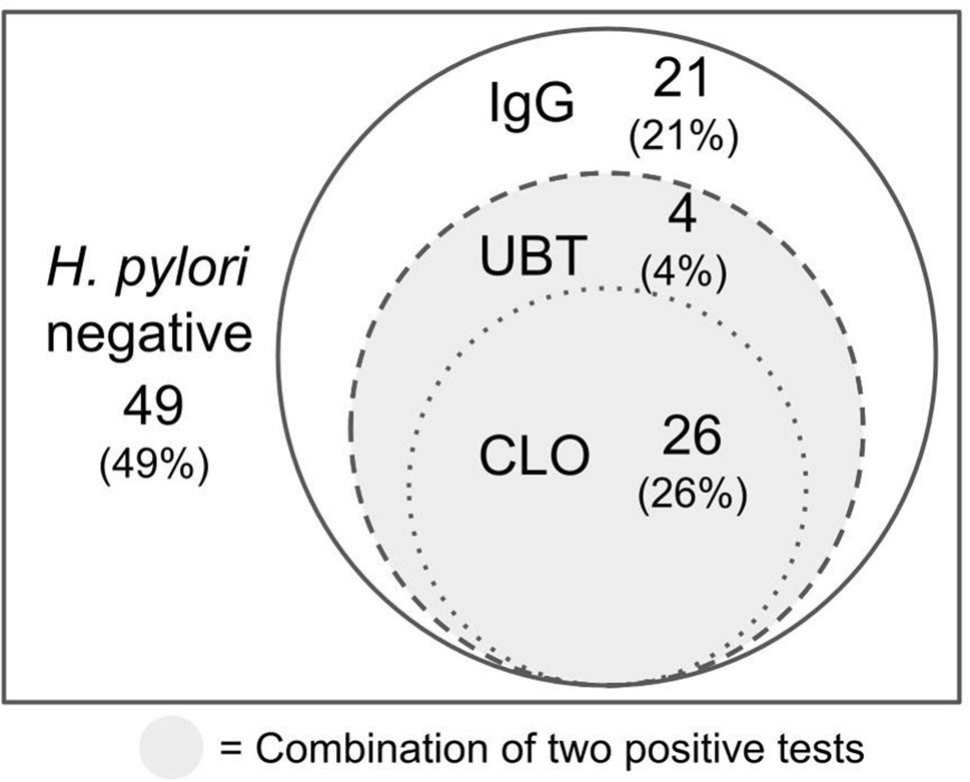
Venn diagram to present the affiliation of each diagnostic test for *H. pylori* infection. A total of 100 patients were enrolled, 49 of them were *H. pylori* infection “triple” negative. The CLO test (CLO) positive patients (n = 26) also had UBT positive and IgG serologic test (IgG) positive. And all UBT-positive patients (n = 30, equals 26 and 4) had IgG test positive. A total of 21 patients only had IgG test positive but UBT and CLO tested negative. The group of a combination of two positive tests overlapped the group of UBT positive.

A total of 100 patients were enrolled, 49 of whom tested negative for *H. pylori* infection in the 'triple' test. Among the patients who tested positive for the CLO test (n = 26), all had positive results in both the UBT and IgG serologic tests. Additionally, all 30 patients who tested positive in the UBT (26 CLO-positive and 4 CLO-negative) also showed positive results in the IgG test. There were 21 patients who only tested positive in the IgG test but had negative results in both the UBT and CLO test. Our data reveals a unique finding where the UBT shared the same positive predictive value (PPV), sensitivity, and specificity with the two positive tests against all the EGD findings. This phenomenon is a result of the same population of patients in both diagnostic methods, as depicted in Fig. 1. The Venn diagram clearly demonstrates that patients with a positive IgG blood test also had a positive UBT result, and those with a positive UBT result also had a positive CLO test. Thus, upon consideration of Youden’s index, our results support the use of UBT as the sole diagnostic tool for *H. pylori* infection, given it demonstrated high sensitivity and high specificity.

### The role of *H. pylori* colonization on different types of gastritis

**Figure 2:**
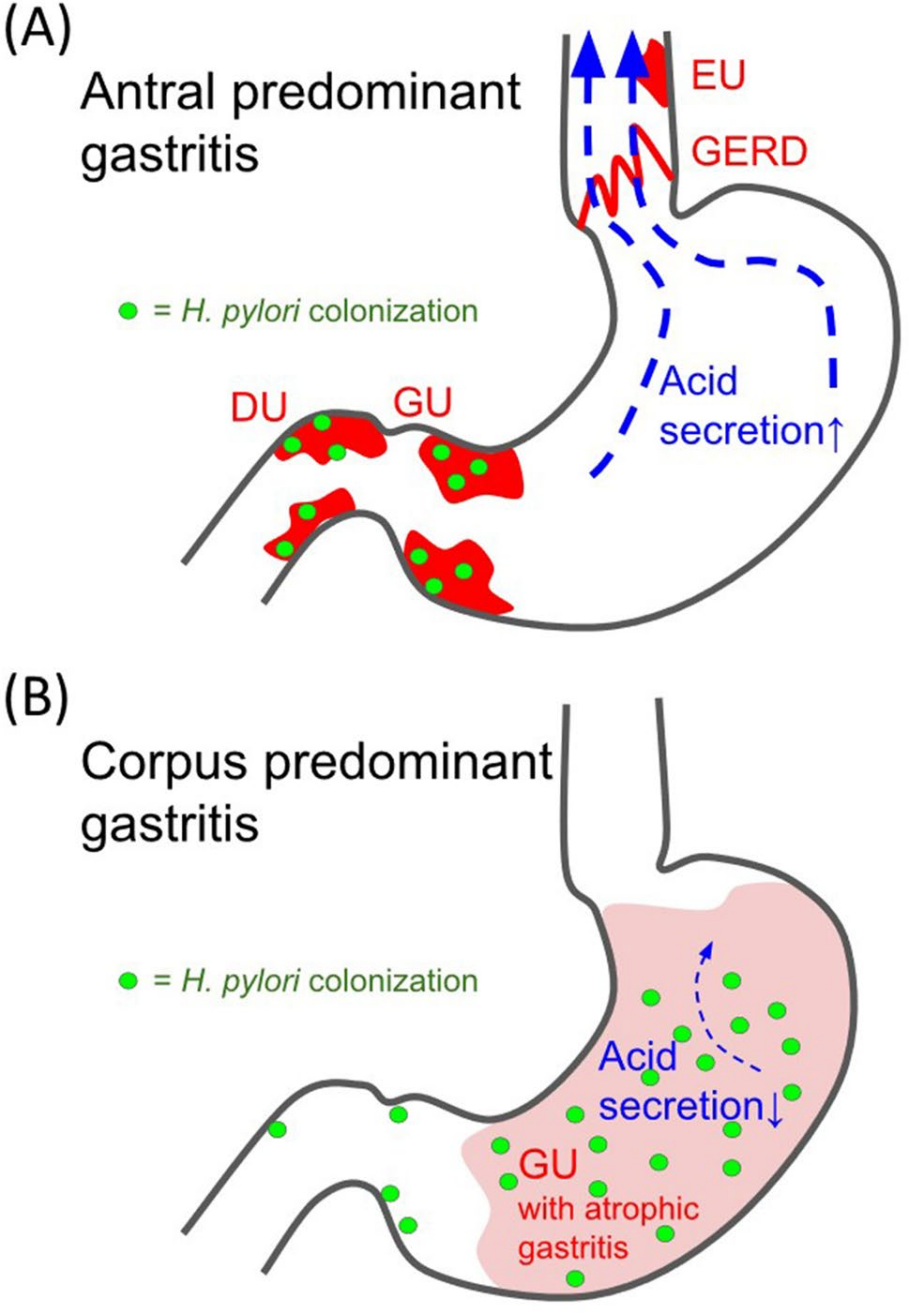
Schematic diagram of antral predominant gastritis and corpus predominant gastritis. The role of *H. pylori* colonization on different types of gastritis. (**A**) *H. pylori*-induced antral predominant gastritis, this pathogen might colonize in the antrum and proximal duodenum. (**B**) *H pylori*-induced corpus predominant gastritis, the inflammatory area might involve almost the whole stomach. Abbreviation: *GU* gastric ulcer, *DU* duodenal ulcer, *GERD* gastroesophageal reflux disease, *EU* esophageal ulcer.

The role of *H. pylori* colonization in different types of gastritis is significant. When the patient has *H. pylori*-induced antral predominant gastritis, this pathogen primarily colonizes the antrum and proximal duodenum, as shown in Fig. 2A. This colonization in specific locations leads to increased acid secretion, resulting in various conditions, including gastric ulcers (GU, especially in the distal stomach), duodenal ulcers (DU), gastroesophageal reflux disease (GERD), and esophageal ulcers (EU). Conversely, when the patient has *H. pylori*-induced corpus predominant gastritis, as illustrated in Fig. 2B, the inflammatory area may involve almost the entire stomach, leading to atrophy due to chronic inflammation. In this case, *H. pylori* colonization might be present throughout the entire stomach, potentially reducing the sampling error of the CLO test. Overall, *H. pylori* colonization plays a crucial role in the development of different types of gastritis, and its location within the gastrointestinal tract can lead to various clinical manifestations.

### Comparisons between each diagnostic test against the findings of esophagogastroduodenoscopy (EGD)

The calculated parameters comparing each diagnostic test to the findings of esophagogastroduodenoscopy (EGD) are presented in Table 4. First, when evaluating for gastroesophageal reflux disease (GERD), the IgG test exhibited a sensitivity of 44.6%, which was higher than all the other tests (ranging from 21.4 to 26.8%). However, its specificity was only 40.9%. For the esophageal ulcers (EU) evaluation, the IgG test displayed the highest sensitivity (44.4%), while the specificity with 45.3%. Although the sensitivity of CLO and UBT for EU evaluation was not high, the specificity of CLO and UBT for EU evaluation was high from 64.1 to 65.6%. When considering gastric ulcers (GU), only the IgG test showed the highest sensitivity (53.1%) and lowest specificity (51.0%), resulting in the lowest Youden's index (0.041). There was high specificity for GU evaluation in the UBT and CLO from 74.5 to 78.4%. Regarding duodenal ulcers (DU), the IgG test exhibited the highest sensitivity (33.3%) and the UBT and CLO exhibited the high specificity from 69.2 to 73.6%. For the evaluation of peptic ulcer disease (PUD), the IgG test displayed the highest sensitivity (50.0%) and UBT or CLO exhibited the highest specificity of 65.4%.

**Table 4:** Calculated parameters of each diagnostic test against EGD finding.

Test/outcome	GERD	EU	GU	DU	PUD
Sensitivity					
IgG	44.6%	44.4%	53.1%	33.3%	50.0%
CLO	21.4%	11.1%	30.6%	22.2%	23.0%
UBT	26.8%	19.4%	34.7%	22.2%	28.4%
Two positive	26.8%	19.4%	34.7%	22.2%	28.4%
Specificity					
IgG	40.9%	45.3%	51.0%	47.3%	46.2%
CLO	68.2%	65.6%	78.4%	73.6%	65.4%
UBT	65.9%	64.1%	74.5%	69.2%	65.4%
Two positive	65.9%	64.1%	74.5%	69.2%	65.4%
Youden's index					
IgG	− 0.145	− 0.103	0.041	− 0.194	− 0.038
CLO	− 0.104	− 0.233*	0.090	− 0.042	− 0.116
UBT	− 0.073	− 0.165	0.092	− 0.086	− 0.062
Two positive	− 0.073	− 0.165	0.092	− 0.086	− 0.062

*IgG* Immunoglobulin G, *CLO* Campylobacter-like organism, *UBT* urea breath test, *GEFD* gastroesophageal reflux disease, *EU* esophageal ulcer, *GU* gastric ulcer, *DU* duodenal ulcer, *PUD* peptic ulcer diseases.

*P < 0.05; **P < 0.001.

The relationships between diagnostic tests and EGD findings were calculated by the Chi-square test (Table 5). Only esophageal ulcer (EU) and CLO test had significantly different P-value (0.011), indicating that a relationship existed between them. Therefore, a binary logistic regression was calculated, and the odds ratio was 0.239 with a significantly different P-value of 0.016, which means the probability of having esophageal ulcer in CLO test positive patients was 0.239 times than in CLO test-negative patients (Table 6).

**Table 5:** P-value of each diagnostic test against EGD finding.

Test/outcome	GERD	EU	GU	DU	PUD
P-value					
IgG	0.151	0.325	0.686	0.266	0.736
CLO	0.240	0.011*	0.303	0.787	0.244
UBT	0.429	0.084	0.315	0.594	0.551
Two positive	0.429	0.084	0.315	0.594	0.551

*P < 0.05; **P < 0.001.

**Table 6:** Binary logistic regression result of diagnostic tests against different EGD findings.

	Beta coefficient	Odds ratio	95% Confidence interval	P-value
IgG to GERD	− 0.583	0.558	0.251–1.242	0.153
IgG to EU	− 0.411	0.663	0.292–1.507	0.326
IgG to GU	0.162	1.176	0.536–2.577	0.686
IgG to DU	− 0.803	0.448	0.106–1.901	0.276
IgG to PUD	− 0.154	0.857	0.350–2.099	0.736
CLO to GERD	− 0.537	0.584	0.238–1.437	0.242
CLO to EU	− 1.433	0.239	0.075–0.762	0.016*
CLO to GU	0.473	1.604	0.651–3.955	0.305
CLO to DU	− 0.226	0.798	0.155–4.108	0.787
CLO to PUD	− 0.574	0.563	0.213–1.490	0.248
UBT to GERD	− 0.346	0.707	0.300–1.670	0.430
UBT to EU	− 0.843	0.089	0.163–1.136	0.089
UBT to GU	0.440	1.553	0.656–3.676	0.317
UBT to DU	− 0.442	0.643	0.126–3.292	0.596
UBT to PUD	− 0.290	0.748	0.289–1.941	0.551

*IgG* Immunoglobulin G, *CLO* Campylobacter-like organism, *UBT* urea breath test, *GEFD* gastroesophageal reflux disease, *EU* esophageal ulcer, *GU* gastric ulcer, *DU* duodenal ulcer, *PUD* peptic ulcer diseases.

*P < 0.05; **P < 0.001.

### Binary logistic regression result of diagnostic tests against different EGD findings

The results of our data collection and Chi-square test analysis indicated a significant relationship between the CLO test and esophageal ulcers. Additionally, binary logistic regression was conducted for each diagnostic test against the findings of esophagogastroduodenoscopy (EGD) (as shown in Table 6). Regardless of the diagnostic tool used, only gastric ulcers exhibited a positive Beta coefficient and an odds ratio greater than 1. This suggests that a positive *H. pylori* test had a weak positive association with gastritis.

## Discussion

As the gold standard for diagnosing *H. pylori* infection remains undefined, researchers have sought to enhance accuracy through various methods. It has been reported that the sensitivity of routine histology, when combined with an additional rapid urease test, can be improved, showcasing the ongoing efforts to refine diagnostic approaches^26^. This suggests that combining the results of at least two diagnostic tests can significantly increase both sensitivity and specificity. Consequently, some studies comparing different diagnostic tools have adopted a combination of two or even three tests as a gold standard^22^. While a study from China demonstrated that combinations of two tests did not provide an additional advantage over the most accurate single test, including histology, CLO test, and UBT^27^. While the non-invasive method, Stool Antigen Test (SAT), is also widely utilized, its inclusion in the study was precluded due to the demanding requirements for stool specimens. Patient compliance becomes challenging in this regard, and suboptimal specimens can significantly compromise accuracy. Consequently, the stool antigen test was not included in the study.

Our data reveals a noteworthy finding: the UBT shares the same sensitivity and specificity as two positive tests against all the Esophagogastroduodenoscopy findings. This phenomenon can be attributed to the same population tested by the two diagnostic methods, as illustrated in Fig. 1. The Venn diagram clearly demonstrates that patients with a positive IgG blood test also include those with a positive UBT, and patients with a positive UBT also include those with a positive CLO test. Consequently, considering Youden’s index, our results support the use of UBT as a single diagnostic tool for *H. pylori* infection due to its sensitivity and specificity.

The association between *H. pylori* infection and esophageal diseases is a controversial issue that had been discussed for decades. The epidemiologic evidence reveals that in Western societies, when the prevalence of *H. pylori* infection decreased, the prevalence of GERD, Barrett’s high-grade dysplasia, and esophageal adenocarcinoma increased^28–30^. Also, one challenge that why around half the world’s population carries this pathogen, but only about 20% of infected people become sick^31^. Thus, researchers assumed that *H. pylori* were a protective factor against esophageal cancer, even mentioned that due to the eradication and lack of *H. pylori* colonization in the stomach, the risk of *Clostridium difficile* infection, as well as contributing to antibiotic resistance, the worldwide epidemic of childhood-onset obesity and diabetes increased^32,33^.

Tracing back to the natural course of gastroesophageal reflux, when esophageal mucosa kept insulting by excessive reflux of gastric acid, esophagitis could be developed, then progressed to Barrett’s dysplasia and finally adenocarcinoma of the esophagus^34^. It seemed reasonable but was not comprehensive enough. Actually, gastroesophageal reflux is a multifactorial problem, related to the balance of harmful factors (such as the acidity of refluxate and esophageal hypersensitivity) and protective factors (a competent esophagogastric junction and esophageal acid clearance)^35^. And *H. pylori* infection seems to only contribute to GERD via gastric refluxate modification through the elaboration of cytotoxic factors^36^, but there is no evidence that *H. pylori* infection can affect esophagogastric junction, or decreases the pressure of the lower esophageal sphincter^37^.

Even though *H. pylori* infection might play a role in gastric reflux, the location and severity of gastritis determined the different effects which was demonstrated in Fig. 2. Antral-predominant gastritis could be induced by the colonization of *H. pylori,* especially in the antrum and proximal duodenum. And this type of gastritis usually resulted in increasing gastric acid secretion, and even duodenal ulcers because the parietal cells were hyper-stimulated by the gastrin and somatostatin-secreting cells due to antral inflammation^38,39^. On the contrary, corpus-predominant gastritis is also known as pangastritis, which means that almost the whole stomach is involved in the inflammation, resulting in decreasing acid production (hypo-chlorhydria). Even total loss of acid secretion (achlorhydria) if the inflammation persisted for a long time, could develop advanced atrophy of oxyntic mucosa^40^.

By comparison of the three current diagnostic tools, the CLO test relied on tissue biopsy, so the mismatched biopsy site and *H. pylori* colonized area which decreased its sensitivity was its major defect^41^. However, if the area of *H. pylori* colonization enlarged such as in the condition of pangastritis, the correct diagnosis rate would increase. Therefore, the potential sampling error turned into a strength instead of the weakness of the CLO test, it might have the capacity to capture the cases of *H. pylori*-induced corpus predominant gastritis compared to other tests. According to our survey, the cost of each test is as follows: CLO test USD 7.42, UBT USD 43.26, and serum *H. pylori* IgG test USD 8.03. Consequently, the UBT stands out as the most expensive test in our hospital. In terms of medical cost-effectiveness, CLO testing proves highly advantageous, especially when the number of tests is substantial.

Since a positive IgG blood test indicates not only current *H. pylori* infection but also previous infection, setting IgG blood test as the reference standard results in the UBT test being unable to capture cases with a previous *H. pylori* infection but without current infection. This dynamic contributes to the observed lower sensitivity. The reason why UBT and IgG blood tests did not yield similar significant findings may be attributed to their respective characteristics. The principle of UBT relies on the capacity of urea hydrolysis by active *H. pylori* in the stomach, detecting isotopically labeled CO_2_ pre-administered into the bloodstream in a breath sample^42^. Consequently, when the pathogen is inactive, it may not be correctly detected. In other words, UBT can capture *H. pylori* infection as long as it is alive and hydrolyzing urea, making its performance less affected by the arrangement of gastritis. On the other hand, the IgG blood test is recommended as an effective tool for assessing the prevalence of *H. pylori* in epidemiological studies. This aligns with our findings, indicating low specificity but high sensitivity^11^. Therefore, regardless of the location of gastritis, both UBT and the IgG blood test should yield positive results. However, a Japanese team found differences in serum antibody titers between patients with varying levels of gastric atrophy^43^. It is worth noting that their study focused on patients with negative anti-*H. pylori* antibody tests but still exhibited low antibody titers, making it not directly comparable to our findings.

Although our results only revealed a significant relationship between the CLO test and esophageal ulcer based on the Chi-square test, each diagnostic test against each EGD finding underwent binary logistic regression, as shown in Table 5. Regardless of the diagnostic tool used, only gastric ulcer exhibited a positive Beta coefficient and odds ratio greater than 1. This suggests that a positive *H. pylori* test had a weak positive relationship with gastritis. This observation could be explained by the persistence of gastric ulcers even as stomach inflammation progressed to severe atrophy, while other findings tended to alleviate due to decreased acid production.

## Conclusion

While UBT incurs a considerable cost, its non-invasive nature, coupled with its high sensitivity and specificity, positions it with significant potential to emerge as the primary diagnostic test for *H. pylori* infection. Additionally, the notable negative correlation observed between the CLO test and esophageal ulcers serves as compelling evidence, underscoring the distinctive effects of *H. pylori* infection on antral-predominant and corpus-predominant gastritis.

### Strengths and limitations of this study

- Comparisons between current diagnostic tools for *H. pylori* infection, including the CLO test, UBT, and IgG blood test.
- Comparisons between each diagnostic test against the EGD finding of *H.pylori* infection.
  - Our sample size was small, and the EGD report should label the influenced area of gastritis, and ulcer, as well as the sampling site of the CLO test.
  - The peptic ulcer disease was not a specific enough category because it enrolled esophageal ulcer and gastric ulcer, which had a contrary correlation to *H. pylori* infection. This category should be divided into at least gastric ulcer and the other for *H. pylori*-related studies.

## Data Availability

The datasets used and/or analyzed during the current study are available from the corresponding author upon reasonable request.
